# Supplementary material for: Comprehensive deletion scan of anti-CRISPR AcrIIA4 reveals essential and dispensable domains for Cas9 inhibition
Source: bioRxiv. 2024 Jul 9:2024.07.09.602757. Preprint. [Version 1] doi: 10.1101/2024.07.09.602757 (PMC11451618; doi:10.1101/2024.07.09.602757)
Supplement: Supplement 1 [file media-1.pdf]

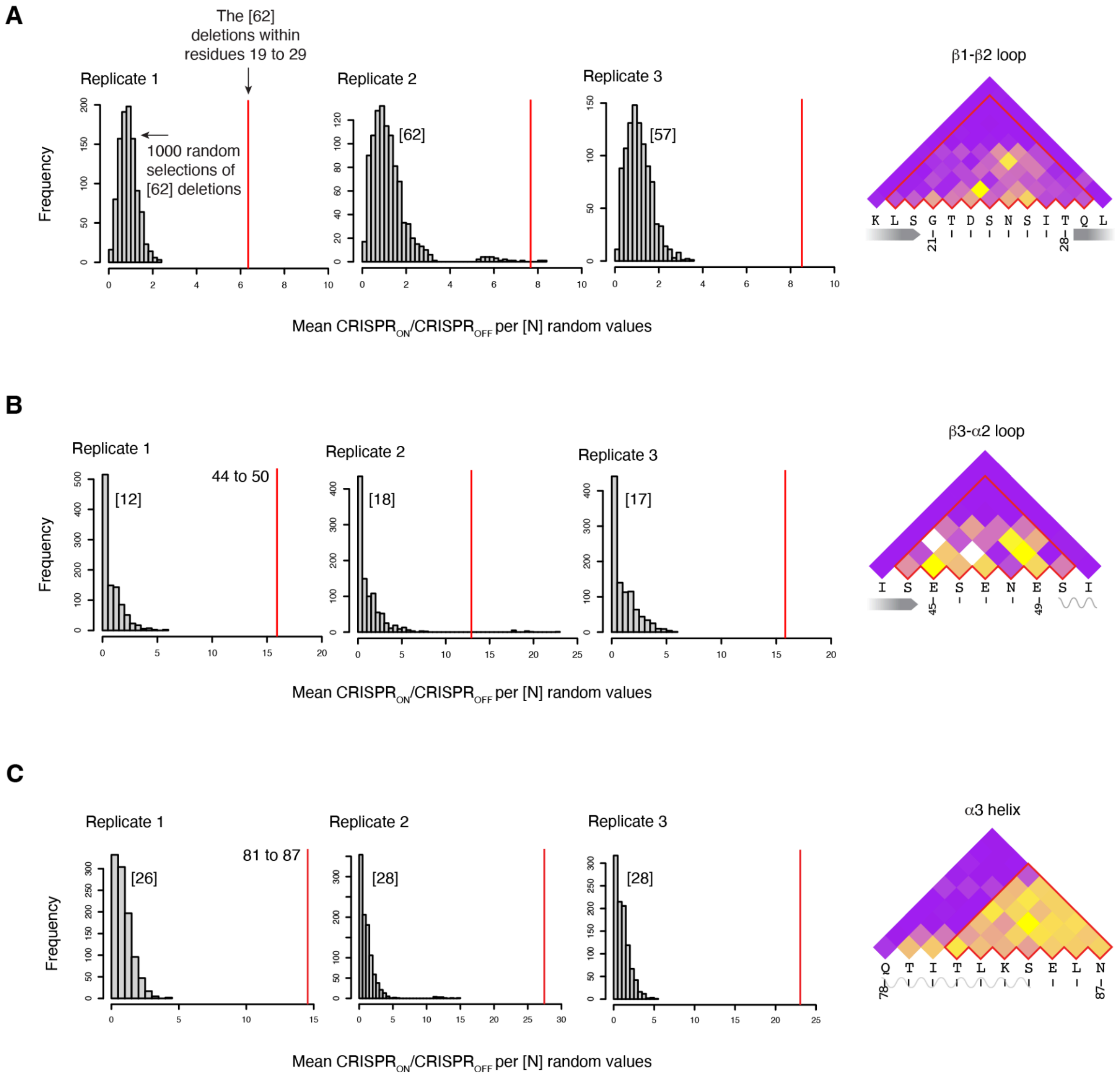

Supplementary Figure 1. Statistical significance of the three dispensable domains. For each replicate, the mean CRISPR$_{ON}$/CRISPR$_{OFF}$ score was taken across the deletions that started and ended within each of three tested domains, and compared to the means of 1000 randomly selected groups of deletions of equal [N] size. The distributions of the means of the randomly selected groups are shown with histograms, while the mean values of the tested domains are indicated with vertical red lines. The selected deletions are visualized on the right as triangular sections, outlined with red, of

the heatmap shown in Fig. 3. Variation in [N] between replicates is due to missing values. (A) The mean of the β1-β2 loop deletion scores in each replicate compared to the means of 1000 random selections of 62 values for Replicates 1 and 2 and 57 values for Replicate 3. (B) Mean of β3-α2 loop deletion scores in each replicate compared to 1000 random selections of 12, 18, and 17 values for Replicates 1, 2, and 3, respectively. (C) The mean of the terminal α3 helix region deletion scores in each replicate compared to 1000 random selections of 26, 26, and 28 values for Replicates 1, 2, and 3, respectively.

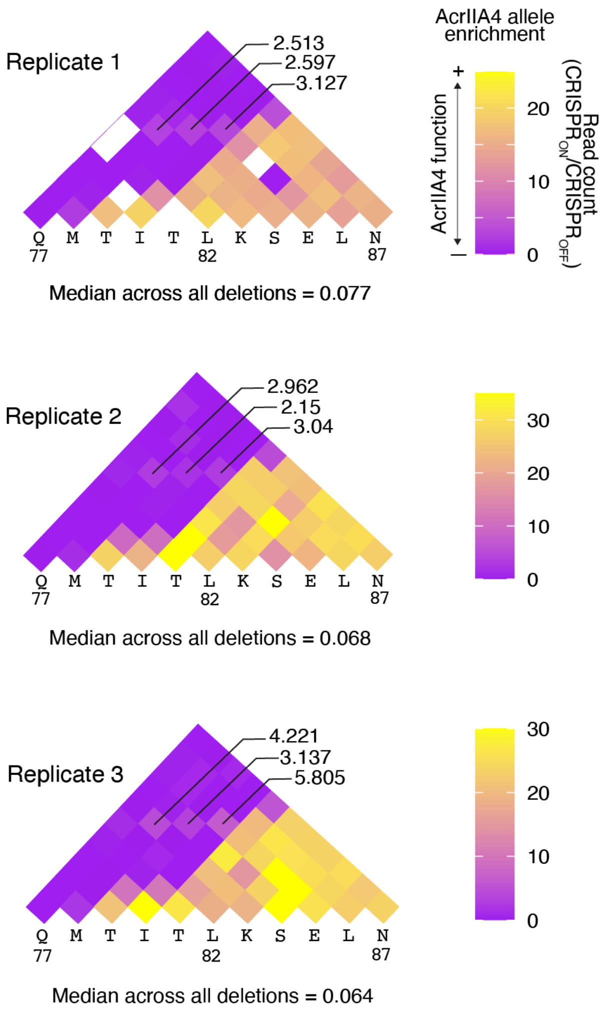

Supplementary Figure 2. Functional scores of the Δ78-83, Δ79-84, and Δ80-85 deletions across the three replicates. Wilcoxon rank-sum tests resulted in p-values < 0.005 for each of the three deletions, comparing their functional scores in the three replicates to the remaining functional scores.

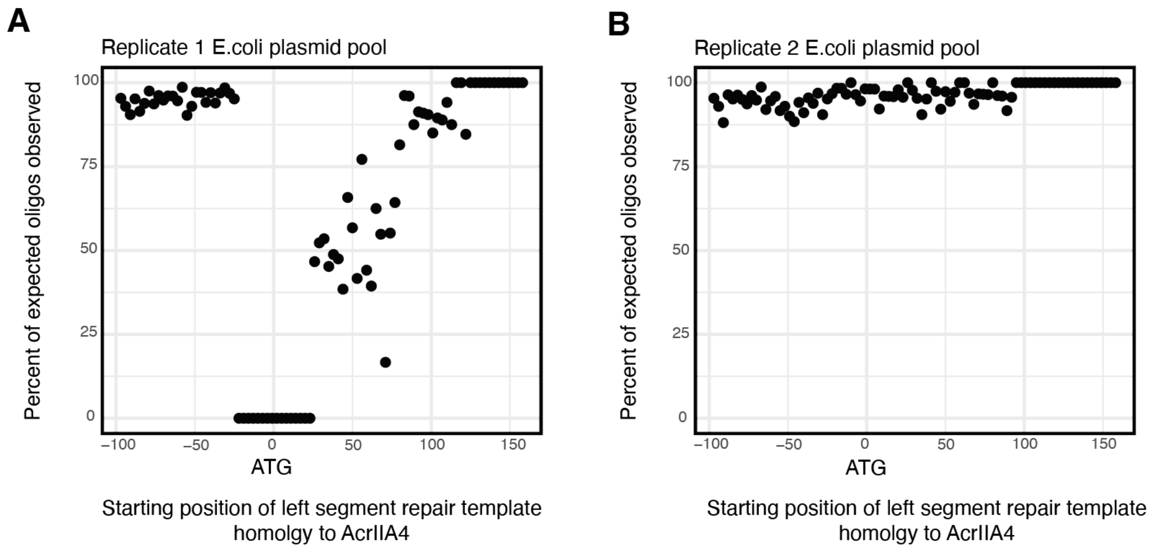

Supplementary Figure 3. *E. coli* plasmid pool sequencing identified an unclone-able sequence that reduced coverage in Replicate 1, which was then removed in Replicate 2. Repair templates are composed of 100 nucleotides of homology to the left of the deletion (the first AcrIIA4 copy) and 100 nucleotides of homology to the right of the deletion (the second AcrIIA4 copy). Starting position refers to the index at which the left-sided homology of a repair template begins relative to the ATG of the first AcrIIA4 copy. (A) Percentage of desired repair templates observed in the sequencing of the *E. coli* plasmid pool of Replicate 1, plotted against the starting position of the repair template. An unidentified issue, likely affecting oligo amplification or cloning efficiency, led to the strong depletion of repair templates whose starting positions ranged from -22 to 23. (B) Resulting *E. coli* plasmid pool of Replicate 2 demonstrating no drop in repair templates whose starting positions ranged from -22 to 23.

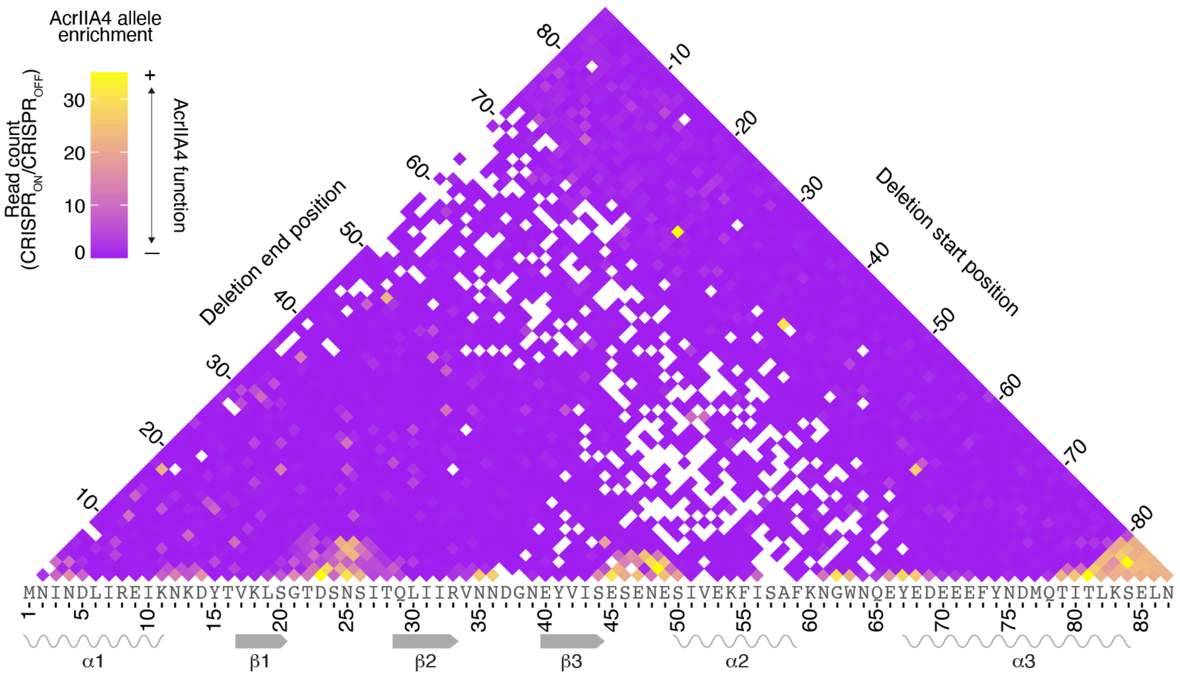

Supplementary Figure 4. Functional characterization of AcrIIA4 alleles determined by their read counts in $CRISPR_{ON}$ conditions normalized against $CRISPR_{OFF}$ read counts. Deletions are colored by the mean value between replicates in which they had at least five reads in glucose, for comparison to the median-base visualization in Fig. 3.

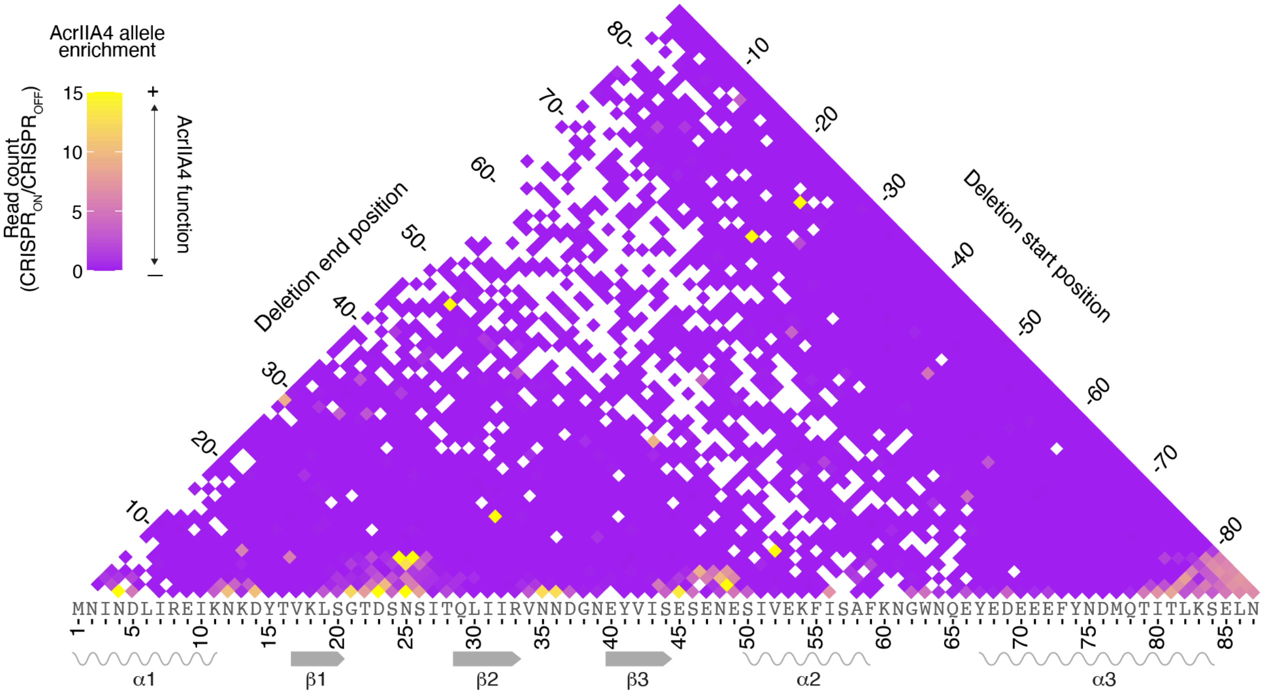

Supplementary Figure 5. Enrichment of repair templates used to generate deletions isolated from CRISPR$_{ON}$ cells relative to CRISPR$_{OFF}$ cells in replicates 2 and 3. Deletions are colored by the minimum value between replicates in which they had at least five reads in glucose. As expected, repair templates that targeted the deletion of dispensable domains were enriched.
